# Acceptability of an alcohol-based handrub gel with superfatting agents among healthcare workers: a randomized crossover controlled study

**DOI:** 10.1186/s13756-022-01129-4

**Published:** 2022-07-15

**Authors:** Alexandra Peters, Jennifer Carry, Charlotte Cave, Julien Sauser, Didier Pittet

**Affiliations:** 1 Infection Control Programme and WHO Collaborating Center on Infection Prevention and Control and Antimicrobial Resistance, University of Geneva Hospitals and Faculty of Medicine, 4 Rue Gabrielle-Perret-Gentil, 1211 Geneva 14, Switzerland; 2 University of Geneva, Geneva, Switzerland; 3 Infection Prevention and Control Department, University Hospital of Nîmes, Nîmes, France

**Keywords:** Hand hygiene, Healthcare workers, Nurses, Tolerability, Alcohol-based handrub, Alcohol-based gel, Infection prevention, Hand sanitizer, Randomized crossover trial, Skin, Intervention

## Abstract

**Introduction:**

Healthcare workers often experience skin dryness and irritation from performing hand hygiene frequently. Low acceptability and tolerability of a formulation are barriers to hand hygiene compliance, though little research has been conducted on what specific types of formulation have higher acceptability than others.

**Objective:**

To compare the acceptability and tolerability of an ethanol-based handrub gel with superfatting agents to the isopropanol-based formulations (a rub and a gel formulation) currently used by healthcare workers at the University of Geneva Hospitals, Geneva, Switzerland.

**Methods:**

Forty-two participants were randomized to two sequences, testing the isopropanol-based formulation that they are using currently (Hopirub® or Hopigel®), and the ethanol-based formulation containing superfatting agents (Saniswiss Sanitizer Hands H1). Participants tested each of the formulations over 7–10 day work shifts, after which skin condition was assessed and feedback was collected.

**Results:**

H1 scored significantly better than the control formulations for skin dryness (*P* = 0.0209), and participants felt less discomfort in their hands when using that formulation (*P* = 0.0448). H1 caused less skin dryness than Hopirub®/Hopigel® (*P* = 0.0210). Though overall preference was quite polarized, 21 participants preferred H1 intervention formulation and 17 preferred the Hopirub®/Hopigel® formulation that they normally used in their care activities.

**Conclusion:**

We observed a difference in acceptability and strongly polarized preferences among the participants' reactions to the formulations tested. These results indicate that giving healthcare workers a choice between different high-quality products is important to ensure maximum acceptability.

**Supplementary Information:**

The online version contains supplementary material available at 10.1186/s13756-022-01129-4.

## Background

Hand hygiene with alcohol-based handrub (ABHR) is the gold standard for most care given in healthcare settings; it prevents both healthcare-associated infections and antimicrobial resistance spread [1, 2]. Good tolerability and acceptability of hand hygiene agents are key to successful adoption of hand hygiene implementation strategies and high compliance [2].

The precise effect of ABHR tolerability and acceptability on healthcare workers (HCWs) compliance has not been quantified, but it is generally considered one of the critical elements. Though there is little published in the literature assessing the link between ABHR tolerability and acceptability to hand hygiene compliance in clinical practice, the World Health Organization (WHO) considers these qualities as prerequisites for any ABHR used in healthcare settings [2]. The WHO had two test protocols concerning ABHR formulations; one is for tolerability, and the second is to help institutions compare tolerability and acceptability of different ABHR formulations [3, 4]. Both isopropanol and ethanol are used in ABHR formulations and there is very little literature about which type of alcohol is best for maximizing skin tolerability and acceptability, though there is increasing evidence for isopropanol’s irritant characteristics on hands with repeated use [5, 6].

HCWs are known to have a high prevalence of dry skin and dermatitis due to their frequent hand hygiene [7]. A number of studies and reviews assessed the levels of skin lesions and contact dermatitis in HCWs both before and during the COVID-19 pandemic [8–11]; the highest prevalence of skin lesions reported occurred in over 80% of HCWs [8]. Providing HCWs ABHR with a high dermal tolerability and high acceptability is crucial for protecting skin intactness so as to not provide an entry point for microbial pathogens, as well as for increasing HCW comfort and compliance. [2]

During the COVID pandemic, the increase in ABHR consumption worldwide resulted in widespread shortages and local production of ABHR formulations [12]. However, some recent studies show that compliance actually decreased during the pandemic, resulting in adverse patient outcomes [13–15]. Other studies showed an increase in compliance during the early phase of the pandemic, and a subsequent decrease to baseline levels as the pandemic progressed [16–18]. Factors as glove use, increased workload, the effect of increased work stress, changes in risk perception, as well as the tolerability and acceptability of the ABHR formulation used may all have an effect.

At the University of Geneva Hospitals (HUG) consumption of ABHR more than doubled between 2019 and 2020 [19]. Hand hygiene compliance improved as well, but only marginally [19]. Though increased consumption by hospital visitors or HCWs taking ABHR home for personal use could explain an increase in consumption without effect on compliance, it is unlikely that this would explain for such a large increase. With respect to the increase in glove use, it is unlikely to positively influence an increase in ABHR use because there is strong evidence in the literature for a negative association between glove use and hand hygiene compliance [20–22]. Internal communication at HUG confirmed an increase in the incidence of adverse skin reactions among HCWs during the pandemic. Though the relationship between ABHR consumption and hand hygiene compliance is complex, tolerability and acceptability warranted further research.

ABHR formulations differ considerably between manufacturers, but there is little published literature comparing the effect of different formulations on tolerability and acceptability [23, 24]. We previously conducted a randomized controlled trial in the laboratory at HUG with 39 participants [25]. Saniswiss Sanitizer Hands H1, Hopigel® and the WHO gel formulation were used for the study. Though there were no differences in the tolerability of the three high-quality ABHRs, there were quite a few differences in acceptability, and participant feedback about which formulations they preferred were strongly polarized [25]. It was therefore decided to assess whether these differences in acceptability carried over to a clinical setting. The objective of this study was to compare the acceptability and tolerability of an ethanol-based ABHR gel with super fatting agents to the isopropanol-based formulations (a rub and a gel formulation) currently used by HCWs at the University of Geneva Hospitals, Geneva, Switzerland.

## Methods

The study assessed the ABHR preferences of HCWs in a randomized crossover design. The formulation chosen for the intervention was Saniswiss Sanitizer Hands H1, which was compared either to Hopirub® or Hopigel® which were the ABHR formulations that the HCWs were using during their work at the time of the study. Hopirub® and Hopigel® are isopropanol-based and also contain chlorhexidine digluconate, while H1 is ethanol-based. H1 contains superfatting agents as the humectant, while Hopigel® and Hopirub® contain isopropyl myristate and bisabolol. All formulations passed the EN1500 standards and are used in healthcare settings.

**Fig. 1:**
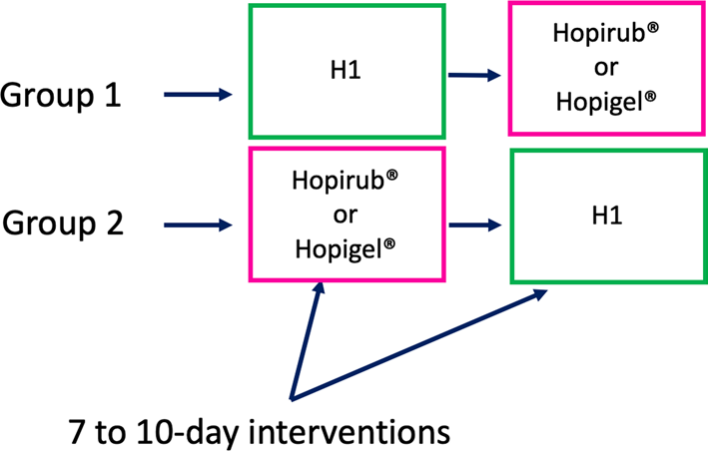
Evaluation of acceptability and tolerability of different alcohol-based handrubs among healthcare workers; randomized crossover controlled study; N = 37 participants

Forty two HCWs from seven wards in the HUG Beau Séjour site (183-bed hospital) participated, and data collection occurred from August 1st to September 15th, 2021. The study design consisted of two consecutive 7 to10-day intervention periods (Fig. 1). The intervention periods were personalized to each participant, and average estimated intervention days were based on individual HCW schedules. Participants were randomized to one of two sequences of formulations; either the ABHR that they were using previously followed by H1 or vice versa. As healthcare workers were already using the control ABHR every day in their work, there was no washout period between interventions. All formulations were prepared in identical bottles with only the coded labels differing; though participants were blinded to the contents of the bottle, they were accustomed to using either Hopirub® or Hopigel®, so it was likely that the majority of them were able to correctly identify which formulation they were testing.

The data collection forms for tolerability and acceptability were adapted from the WHO “Protocol for Evaluation and Comparison of Tolerability and Acceptability of Different Alcohol-based Handrubs: Method 2” [4]. HCW participation was voluntary and participants were included if their employment was 70% at minimum. HCWs were only allowed to participate if they had less than three weeks of vacation during the study period, and were using either Hopirub® or Hopigel®.

Individual appointments were made with all participants to take baseline measurements and distribute the bottles of ABHR. Skin type was determined by color in accordance with the WHO protocol [4]. Researchers collected participant data including: ward, sex, age, number of years of experience and estimated frequency of ABHR use. Baseline evaluations collected additional participant data including: skin color, activities that might impact skin condition, hand cream use and history of dermatitis. HCWs used pocket-sized 100 mL bottles filled with the test formulations instead of the typical pocket-sized bottles that are used at HUG since 1995 [26]. After a 7 to 10-day intervention period, researchers met with each HCW for the follow up evaluation and semi-structured interview (Additional file 1: Appendix: Assessment of tolerability to alcohol-based handrubs, translated from French). If the end of the first intervention coincided with the beginning of the second intervention, the first follow-up and the second baseline coincided. If the HCW was on vacation between the two intervention phases, the second baseline evaluation was performed upon their return to work.

The primary outcome was to assess whether the differences in acceptability and the polarization of preferences observed in the laboratory study [25] would be replicated in a clinical setting. The secondary outcome was the difference in skin tolerability from baseline. To evaluate acceptability, participants gave feedback on the test formulation’s qualities of color, smell, texture, stickiness, presence of deposits or threads, irritation, drying effect, ease of use, speed of drying, application, and overall evaluation. The form used was based on the WHO protocol for assessing tolerability and acceptability (Method 2) [4]. Acceptability, participant preference and any additional feedback was collected in a short semi-structured interview after each intervention.

For the secondary outcome, tolerability was measured by two methods: assessment by a trained observer and self-assessment by the HCW. Observers evaluated HCWs’ skin for redness, scaliness and fissures [4]. Redness ranged from 0 to 4, scaliness from 0 to 5 and fissures from 0 to 3, zero being an absence of symptoms and the increasing numbers relating to the increasing severity of symptoms. Participants self-reported their skin condition from 0 to 4 for the following elements: appearance, integrity, hydration level and sensations (Appendix).

Tolerability measurements were taken at baseline and after the interventions (Table 3). Intention-to-treat population was used for the analyses including all participants randomized having completed at least one of the intervention periods, meaning that the population studied included 37 participants. Descriptive statistics reported mean standard deviation (SD) or median and interquartile range [IQR] as appropriate. Estimated intervention differences were assessed between the intervention (H1) and control (Hopirub® or Hopigel®) with 95% confidence intervals (CIs). The comparisons between interventions used paired t-tests. To assess the robustness of the analyses to the normality assumptions, non-parametric Wilcoxon signed-rank tests were applied. No correction for multiplicity was used. All statistical analyses were performed using R version 3.6.3 or greater.

This study did not fall within the framework of the human research act of the 30 September 2011 (HRA, SR 810.30), but concerned care practices already in use at HUG, so no approval by the ethics committee was needed. The approval for the study was the responsibility of the Academic Council of HUG. An informed consent form was given to each participant containing the information necessary to make a decision regarding their participation in the study. Each participant was informed that their participation in the study was voluntary and that they could withdraw at any time. Confidentiality was guaranteed by the use of participant identification code numbers corresponding to the previously determined randomization list. The data is property of University of Geneva Hospitals and Faculty of Medicine.

## Results

All participating HCWs completed a previously used and tested questionnaire on hand hygiene and personal habits [27]. Forty-two volunteers were recruited to complete the trial, of which 41 were nurses or nursing assistants; 37 completed both intervention periods. Five participants were not included in the study, dropping out due to scheduling conflicts, changing their workplace, or their data was not included due to non-compliance with the intervention. For the baseline analysis intention-to-treat was used, and for the subsequent analysis per protocol set was used and only compliant participants who tested both formulations were included in the final analysis.

Each of the 37 HCWs who completed the study averaged 9.67 days of interventions; 9.74 (1.09) days in the Hopirub® /Hopigel® control arm; and 9.60 (2.17) days in the H1 arm of the study. Though there was a mixture of shift durations at HUG during COVID, the majority of HCWs worked 8-h shifts. A total of 37 (88.10%) of the initially recruited participants were female; the median age was 39 years (30.2–51.8). Of the participants who completed the full questionnaire, 39/40 (97.50%) reported using ABHR more than 25 times per day, and 24/40 (60.00%) reported using ABHR more than 50 times per day. Twenty-four out of 40 (60.00%) participants reported having skin problems; and of those who did, 19/24 (79.16%) reported ABHR as the reasons for their skin problems. The same number and percentage of HCWs reported that these skin problems affected their work at least some of the time, and 10/40 (25%) changed ABHR products in the past due to their skin condition. Data on skin color, activities that might impact skin condition, hand cream use and history of dermatitis were recorded (Table 1). In line with the aforementioned literature, HCWs in this study had a higher incidence of dermatitis than the sample from the general population observed in the previous laboratory study we conducted [25]. Non-parametric Wilcoxon signed-rank tests were performed, and they confirmed our overall results.

**Table 1:** Acceptability of an alcohol-based handrub gel with superfatting agents among healthcare workers: participant characteristics; randomized-crossover study; N = 42

Age	Median	39	
	Mean	40.8 (12.2)	
	Q1–Q3	30.2–51.8	
		Number	Percentage
Sex (n = 42)	Female	37	88.10
	Male	5	11.90
Type of skin (n = 42)	very fair with freckles	4	9.52
	fair ± freckles	13	30.95
	light brown	15	35.71
	brown	5	11.90
	dark brown	1	2.38
	black	4	9.52
Frequency of ABHR use per shift (n = 40)	< 25	1	2.50
	25–50	15	37.50
	51–75	22	55.00
	> 75	2	5.00
Presence of skin issues (n = 40)	Yes	24	60.00
	No	16	40.00
Skin issues due to ABHR (n = 24)	Yes	19	79.17
	No	5	20.83
Skin condition affects work (n = 24)	Not at all	5	20.83
	Sometimes	14	58.33
	Often	5	20.83
Dermatitis diagnosed by physician (n = 40)	Yes	6	15.00
	No	34	85.00
Changed ABHR due to skin issues (n = 40)	Yes	10	25.00
	No	30	75.00

### Acceptability

As the primary outcome was the difference in acceptability between the formulations, participants who only tested one of the two formulations were excluded from the analysis. The mean and standard deviation (SD) for the formulations tested are detailed in Table 2. As shown, better acceptability score was observed for H1 than Hopirub®/Hopigel® for all the elements, except for stickiness and residue; a lower score indicating a better acceptability. Importantly none of the differences observed reached statistical significance except dryness (Table 2). For the sum of all the elements (total score); H1 had a lower total score than Hopirub®/ Hopigel®, indicating that H1 had better acceptability. The average and overall evaluation categories were quite close, with the average of the individual elements scoring slightly better for both groups than the score participants assigned to the overall evaluation. The overall evaluation for H1 was 1.16 (1.42) and it was 1.76 (1.74) for Hopirub®/ Hopigel®. The average of the sum of the individual elements (not including the overall evaluation) was 0.77 for H1 and 0.98 for Hopirub®/ Hopigel®. Participants preference for smell approached the traditional threshold of significance; HCWs preferred the smell of the H1 formulation compared Hopirub®/Hopigel® (*P* = 0.054). H1 caused less skin dryness than Hopirub®/Hopigel® (*P* = 0.021) (Table 2). Though overall preference was quite polarized, 21 participants preferred H1 intervention formulation and 17 preferred the Hopirub®/Hopigel® formulation that they normally used in their care activities.

**Table 2:** Acceptability of each formulation as scored by healthcare workers*; differences in acceptability between intervention and control alcohol-based handrub formulations, paired-student test;** randomized-crossover study, N = 37 participants

	H1	Hopirub/gel	H1–Hopirub/gel	
	Mean (SD)	Mean (SD)	Estimate (IC 95%)	*P* value
*Acceptability*				
Color	0.11 (0.46)	0.19 (0.62)	− 0.081 (− 0.226; 0.063)	0.2625
Smell	0.97 (1.54)	1.73 (2.04)	− 0.757 (− 1.528; 0.015)	0.0543
Texture 1: Pleasantness	0.95 (1.33)	1.08 (1.57)	− 0.135 (− 0.918; 0.647)	0.7282
Texture 2: Stickiness	1.08 (1.38)	0.95 (1.63)	0.135 (− 0.607; 0.877)	0.7140
Texture 3: Residue or threads	0.60 (1.19)	0.46 (1.19)	0.135 (− 0.438; 0.708)	0.6353
Irritation	0.97 (1.52)	1.62 (1.89)	− 0.649 (− 1.424; 0.126)	0.0982
Dryness	1.03 (1.26)	1.95 (1.93)	− 0.919 (− 1.690; − 0.147)	0.0210
Ease of application	0.16 (0.44)	0.30 (0.74)	− 0.135 (− 0.420; 0.150)	0.3428
Speed of drying	1.03 (1.50)	1.38 (1.82)	− 0.351 (− 1.176; 0.474)	0.3935
Overall	1.16 (1.42)	1.76 (1.74)	− 0.595 (− 1.468; 0.279)	0.1758
Total Score	6.89 (5.75)	8.85 (8.25)	− 2.676 (− 6.265; 0.914)	0.1393

*Acceptability was scored 0–7 and is expressed as mean ± SD (standard deviation); total score was calculated on a scale of 0–63, and does not include the general score; lower scores indicate better acceptability. **A negative difference indicates that H1 scored better than the control (Hopirub/gel)

### Skin tolerability: self-evaluation

Participants were asked to evaluate their skin’s appearance, integrity, level of hydration and physical sensations on a scale of 1–7, and the total was calculated; lower values indicated better self-evaluation (Table 3). Participants were asked whether their hands were in better or worse condition than usual. The H1 formulation garnered the most positive self-evaluation with an improvement in skin condition for all elements between baseline and follow-up; with the exception of oedema, which showed no change (Table 3). The Hopirub®/Hopigel® arm showed a slight worsening from baseline for all scored elements (Table 3). The only statistically significant difference for changes from baseline between the intervention and control arms was that H1 decreased discomfort in participants’ hands (Table 3).

**Table 3:** Change in skin tolerability as a mean of the difference between intervention and baseline; differences in tolerability between intervention and control alcohol-based handrub formulations as assessed by healthcare worker self-evaluation* and observer evaluation; paired-student test;** randomized-crossover study; N = 37 participants

	H1	Hopirub/gel	H1–Hopirub/gel	
	Mean (SD)	Mean (SD)	Estimate (IC95%)	*P* value
*Self-evaluation*				
Redness	− 0.15 (0.66)	0.24 (0.91)	− 0.351 (− 0.818; 0.115)	0.1353
Skin rash	− 0.03 (0.42)	0.05 (0.46)	− 0.081 (− 0.295; 0.132)	0.4461
Oedema	0.00 (0)	0.08 (0.36)	− 0.081 (− 0.202; 0.040)	0.1833
Fissures	− 0.18 (0.64)	0.21 (0.87)	− 0.405 (− 0.886; 0.076)	0.0960
Desquamation	− 0.13 (0.79)	0.26 (1.06)	− 0.405 (− 0.964; 0.153)	0.1495
Dryness	− 0.33 (1.12)	0.08 (1.40)	− 0.351 (− 1.085; 0.383)	0.3381
Itching	− 0.10 (0.67)	0.13 (1.07)	− 0.243 (− 0.755; 0.269)	0.3415
Burning	− 0.08 (0.66)	0.24 (0.82)	− 0.270 (− 0.712; 0.172)	0.2231
Pain	− 0.28 (0.85)	0.29 (0.87)	− 0.595 (− 1.175; − 0.015)	0.0448
*Observer evaluation*				
Redness	− 0.05 (0.45)	0.08 (0.27)	− 0.135 (− 0.360; 0.089)	0.2301
Scaliness	− 0.23 (1.07)	− 0.39 (1.00)	0.270 (− 0.272; 0.813)	0.3190
Fissures	0.00 (0)	0.03 (0.16)	0 (NA)	NA

*Participants were asked to evaluate their skin’s appearance, integrity, level of hydration and physical sensations on a scale of 1–7, and the total was calculated; lower values indicated better self-evaluation.

**A negative difference indicates that H1 scored better than the control (Hopirub/gel)

### Skin tolerability: objective assessment

Table 3 shows the differences in skin tolerability assessed by observers between baseline and follow-up after the use of the test formulations. H1 was shown to reduce redness and scaliness while having no impact on fissures, and Hopirub®/Hopigel® slightly increased redness and fissures, but reduced scaliness. The analysis did not show any statistically significant differences between the two groups of formulations (Table 3).

## Discussion

Though H1 was more often preferred over Hopirub®/Hopigel®, and performed better in some of the analyses, the differences in acceptability between the formulations may have been underestimated in the study due to a number of factors. At HUG, HCWs who have skin reactions that are severe enough to be seen by a physician may choose to use another ABHR formulation provided by the hospital. Therefore, the HCW population whose skin did not tolerate Hopirub®/Hopigel® were excluded from participating in the study. Secondly, because HCWs are already allowed to choose between Hopirub® and Hopigel® for their daily care activities, and there was such a high prevalence skin problems due to ABHR that 25% or participants had already changed formulations; there is a strong chance that participants had already chosen the formulation that worked best for them, and were comfortable and accustomed to that formulation. Notably, participants with all types of skin color had a variety of skin problems due to ABHR. This indicates that although it is part of the WHO tool, and may be important when selecting a formulation, skin color may not be a determinant for whether a person’s skin can easily tolerate ABHR formulations or not.

Overall feedback from HCWs was very positive, and the majority of participants were active and engaged when completing the baseline and follow-up assessments and providing their feedback to the research team. Although all formulations were provided in unmarked bottles and participants were theoretically blinded to the intervention, they were very familiar with the formulation they used during routine care, and a number of them were aware of which formulation they were testing. Nevertheless, it was clear from some of the follow-up interviews that this was not always the case.

Concerning the acceptability scoring, the two elements of texture where H1 scored worse than the control formulations (stickiness and residue) could be explained by the fact that Hopirub® is a rinse formulation, not a gel formulation. Therefore, it has no stickiness or residue whatsoever, and thus reduced the mean stickiness and residue score of the Hopirub®/Hopigel® control group.

Because the study took place during the summer vacation period, accurate measurements of formulation tolerability, especially by the observer, were not completely possible as intervention periods were often interrupted by days off. In order to minimize such a confounder, intervention periods were selected according to HCWs’ schedules and arranged to be as close to 10 days as possible, taking into account the timing and the number of days off. Efforts were made to conduct the follow up assessment after a maximum number of intervention days; i.e. preference was given to a slightly shorter intervention rather than to performing follow-up measurements directly after a number of days off. This also resulted in that for some participants the follow up for the first intervention arm and the beginning of the second intervention arm coincided on the same day, while others completed their second baseline measurements after their days off. It is possible that the participant self-evaluation for tolerability may have been more accurate than the observer evaluation as HCWs could observe their hands every day.

Though unused bottles were collected by researchers after the intervention in order to measure the volume as a surrogate for hand hygiene compliance, an accurate assessment of the volume of each formulation used was not possible. Because researchers gave HCWs all bottles for 10 days at one time, they relied on participant memory and their participation in returning the unused bottles of test formulations, and numerous HCWs did not return all their bottles.

The external validity of these findings remains limited for acceptability and tolerability of these specific formulations because of the limitations concerning the small sample size, issues with scheduling the intervention periods, and the fact that most healthcare facilities will not be comparing these specific products. Nevertheless, we believe that the finding that HCWs have strongly polarized preferences will be applicable to other settings and with other ABHRs. Our previous study conducted in the laboratory further supports this hypothesis [25]. We interpret these findings that in order to increase HCW satisfaction and possibly increase compliance, HCWs should be given a choice of ABHR formulations.

## Conclusion

The results from this study involving healthcare personnel working on multiple wards support the initial results from the study conducted in the laboratory. H1 performed slightly better overall and was preferred more often than Hopirub®/Hopigel®. It is increasingly clear that acceptability is a very individual issue, and that HCWs should be given a choice between products, preferably ones that use different types of alcohols and different types of moisturizing agents. Though it seems highly probable that acceptability, tolerability, and compliance are linked given the frequency of painful skin conditions associated with high exposure to ABHR use, more research needs to be performed to better understand this relationship. Additional research is also needed to determine whether HCW hand hygiene compliance increases significantly when an individual uses an ABHR that has a high acceptability for them.

## Supplementary Information

**Additional file 1.** Assessment of tolerability to alcohol-based handrubs.

## Data Availability

All data generated or analyzed during this study are included in this published article, as supplementary files, or available on request.
