# Supplementary material for: Acceptability of an alcohol-based handrub gel with superfatting agents among healthcare workers: a randomized crossover controlled study
Source: Antimicrob Resist Infect Control. 2022 Jul 15;11:97. doi: 10.1186/s13756-022-01129-4 (PMC9283849; doi:10.1186/s13756-022-01129-4)
Supplement: Supplementary file 1 — Additional file 1. Assessment of tolerability to alcohol-based handrubs. [file 13756_2022_1129_MOESM1_ESM.docx]

**Assessment of tolerability to alcohol-based handrubs**

Adaptation and translation from*: WHO Protocol for Evaluation of Tolerability and Acceptability of Alcohol-based Handrub in Use or Planned to be Introduced: Method 1*

|  |
| --- |

Date : Participant number:

Product : A / B Weight of the bottles :

*Assessment of factors influencing skin tolerance*

*You should answer the following questions based on the week before this questionnaire.*

|  | Yes 1 |  | No 0 |
| --- | --- | --- | --- |

Have you recently participated in any activity(ies) outside of work that may have caused damage to your skin?

|  | Gardening | 1 |
| --- | --- | --- |
|  | Do-it-yourself | 2 |
|  | Cleaning | 3 |
|  | Sport | 4 |
|  | Other : | 5 |

If yes, which activity ?

Do you use a moisturizing hand cream or lotion ?

|  | Never | 0 |
| --- | --- | --- |
|  | Rarely | 1 |
|  | Sometimes, depending on the season | 2 |
|  | Once per day | 3 |
|  | Several times per day | 4 |

|  | Yes 1 |  | No 0 |
| --- | --- | --- | --- |

Do you have any other comments ?

*Self-assessment by the participant of the condition of his/her skin today*

*You should answer the following questions based on the condition of your skin as you see it now. Circle the number that you think best fits.*

|  | **Not at all** | | **A little** | **Somewhat** | **A lot** | **Everywhere** |
| --- | --- | --- | --- | --- | --- | --- |
| *Skin appearance* |  | |  |  |  |  |
| Redness | 0 | | 1 | 2 | 3 | 4 |
| Rash | 0 | | 1 | 2 | 3 | 4 |
| Oedema | 0 | | 1 | 2 | 3 | 4 |
| *Skin integrity* |  | |  |  |  |  |
| Cracks / Abrasions | 0 | | 1 | 2 | 3 | 4 |
| Scaliness (flaking) | 0 | | 1 | 2 | 3 | 4 |
| *Skin hydratation level* | |  |  |  |  |  |
| Dryness | 0 | | 1 | 2 | 3 | 4 |
| *Sensations* |  | |  |  |  |  |
| Itching | 0 | | 1 | 2 | 3 | 4 |
| Burning | 0 | | 1 | 2 | 3 | 4 |
| Pain | 0 | | 1 | 2 | 3 | 4 |

|  | Better -1 |  | The same 0 |
| --- | --- | --- | --- |

|  | Worse 1 |
| --- | --- |

If your skin condition has changed, is it better or worse than normal?

|  | Yes 1 |  | No 0 |
| --- | --- | --- | --- |

Do you have any other comments ?

*Participant assessment of the product (acceptability)*

*You should rate the product you have been given by circling the number that you think best fits. The numbering ranges from best (=0) to worst (=6).*

| *Color :* |  |  |  |  |  |  |  |  |
| --- | --- | --- | --- | --- | --- | --- | --- | --- |
| Pleasant | 0 | 1 | 2 | 3 | 4 | 5 | 6 | Unpleasant |
| *Smell :* |  |  |  |  |  |  |  |  |
| Pleasant | 0 | 1 | 2 | 3 | 4 | 5 | 6 | Unpleasant |
| *Texture :* |  |  |  |  |  |  |  |  |
| Pleasant | 0 | 1 | 2 | 3 | 4 | 5 | 6 | Unpleasant |
| Not sticky | 0 | 1 | 2 | 3 | 4 | 5 | 6 | Sticky |
| No lumps/ threads | 0 | 1 | 2 | 3 | 4 | 5 | 6 | Lumps/threads |
| *Irritation :* |  |  |  |  |  |  |  |  |
| Not irritating | 0 | 1 | 2 | 3 | 4 | 5 | 6 | Irritating |
| *Dryness:* |  |  |  |  |  |  |  |  |
| Soft/ flexible | 0 | 1 | 2 | 3 | 4 | 5 | 6 | Dry/ rough |
| *Ease of use :* |  |  |  |  |  |  |  |  |
| Easy | 0 | 1 | 2 | 3 | 4 | 5 | 6 | Difficult |
| *Speed of drying :* |  |  |  |  |  |  |  |  |
| Fast | 0 | 1 | 2 | 3 | 4 | 5 | 6 | Slow |
| *Overall evaluation :* |  |  |  |  |  |  |  |  |
| Pleasant | 0 | 1 | 2 | 3 | 4 | 5 | 6 | Unpleasant |

|  | Yes 1 |  | No 0 |
| --- | --- | --- | --- |

Do you have any other comments ?

*Investigator's assessment of the skin tolerance of the participant* ***today***

*An investigator will is assessing the skin condition of your hands based on a previously established visual score. Scaliness is calculated as the mean of the severity and distribution of skin scale.*

|  | **No redness** | **Slight redness or**  **blotchiness** | **Moderate redness** | **Bright red** | **Bright red with oedema present** | |
| --- | --- | --- | --- | --- | --- | --- |
| *Redness* | 0 | 1 | 2 | 3 | 4 | |
|  | **Normal** | **Very slightly scaly** | **Slightly scaly** | **Scaly** | **Scaly to very scaly** | **Very scaly** |
| *Scaliness** | 0 | 1 | 2 | 3 | 4 | 5 |
|  | **No fissures** | **Very fine** | **Large, either single or multiple** | **Extensive cracks with bleeding or seeping** | | |
| *Fissures* | 0 | 1 | 2 | 3 | | |

** Visual scoring of the severity of skin scale:*

| **Normal** (no observable scale or irritation of any kind) | 0 |
| --- | --- |
| **Very slightly scaly** (occasional scale) | 1 |
| **Slightly scaly** (scale in sulci and on plateaus, no wide-spread uplifting) | 2 |
| **Scaly** (visible scale may give the skin surface a whitish appearance**.** Definite uplifting of edges or scale-sections. Skin is rough to the touch) | 3 |
| **Scaly to very scaly** (more scale and pronounced separation of scale edges from skin, although they may still be lying flat on the skin surface. Some evidence of cracking in sulci and on plateaus. Also, skin may appear irritated with some reddening) | 4 |
| **Very scaly** (Cracking of skin surface. In some cases, scales are very large, but some individuals never develop large scales. The skin may appear to be very irritated, with wide-spread reddening and/or occasional bleeding) | 5 |

** Visual scoring of the distribution of skin scale:*

| **Normal** (no observable scale or irritation of any kind) | 0 |
| --- | --- |
| **Very limited distribution** (less than 10 % of hand surface) | 1 |
| **Limited distribution** (less than 20% of hand surface) | 2 |
| **Significant distribution** (20-50% of hand surface) | 3 |
| **Widely distributed** (50-75% of hand surface) | 4 |
| **Total distribution** (75-100% of hand surface) | 5 |
